# Dose–response relationship between cotinine levels and female reproductive lifespan

**DOI:** 10.1186/s41043-025-01229-y

**Published:** 2026-01-16

**Authors:** Jie Liao, Tingting Liu, Aijie Xie, Xunmei Zhou, Xin Li, Hengxi Chen

**Affiliations:** 1 Tibet Autonomous Region Women’s and Children’s Hospital, Second University Hospital of Sichuan University, West, Lhasa, China; 2 West China Second University Hospital, Sichuan University, Chengdu, 610041 China; 3 Key Laboratory of Birth Defects and Related Diseases of Women and Children, West China Second Hospital, Sichuan University, Ministry of Education, Sichuan University, Chengdu, 610041 China; 4 School of Medicine, Chengdu Women’s and Children’s Central Hospital, University of Electronic Science and Technology of China, Chengdu, 611731 China

**Keywords:** Cotinine, Reproductive lifespan, Age at menopause, Tobacco exposure, NHANES, KNHANES

## Abstract

**Background:**

Tobacco exposure is a major public health concern and has been implicated in accelerated female reproductive aging. However, most evidence relies on self-reported smoking history, which may introduce bias. Cotinine, a reliable biomarker of nicotine exposure, provides an objective measure to clarify the association between tobacco exposure and reproductive lifespan (RLS).

**Methods:**

We analyzed 11,944 women from two nationally representative cohorts: NHANES (*n* = 6,081, U.S., 1999–2018) and KNHANES (*n* = 5,863, Korea, 2014–2020). Serum cotinine (NHANES) and urinary cotinine (KNHANES) were quantified using standardized laboratory assays. Multivariable linear regression and restricted cubic spline (RCS) models were employed to assess the relationship between cotinine levels and age at menopause, menarche, and RLS, adjusting for demographic, socioeconomic, and metabolic covariates. Subgroup analyses were conducted to explore effect modification.

**Results:**

Higher cotinine levels were significantly associated with earlier menopause (NHANES β = −0.23; KNHANES β = −0.10) and shorter RLS (NHANES β = −0.22; KNHANES β = −0.08). RCS models confirmed linear dose–response associations in both cohorts, with threshold effects observed at higher exposure levels (NHANES ln-cotinine > − 3.47: β = −0.303, 95% CI: −0.386 to − 0.220, *P* < 0.001). Subgroup analyses indicated stronger associations among younger women, non-diabetic individuals, and lower-income groups, with pronounced differences across racial and educational strata.

**Conclusions:**

Cotinine, as an objective biomarker of tobacco exposure, was robustly associated with shortened reproductive lifespan across two national cohorts. The associations were linear, with stronger reproductive toxicity at higher exposure levels, particularly among U.S. women. These findings highlight the reproductive risks of smoking and underscore the importance of biomarker-based assessments in reproductive aging research.

## Backround

Tobacco use has been recognized as a significant global public health challenge, and its damage to the cardiorespiratory system has long been well documented [1, 2]. However, the potential harmful effects of tobacco exposure on the female reproductive system have not received sufficient attention. Existing studies have shown that smoking not only reduces fertility and affects pregnancy outcomes [3] but may also lead to early menopause [4]. On average, women who smoke enter menopause approximately 1 year earlier than non-smokers [5], and women exposed to cigarette smoke have significantly reduced ovarian volume [6], suggesting that tobacco exposure may accelerate the process of reproductive aging through multiple mechanisms.

The age of menarche and menopause defines reproductive lifespan (RLS) in women and reflects the dynamic process of ovarian function from development to decline [7]. As an indicator that encompasses both the initiation and decline processes of ovarian function, reproductive lifespan has a higher overall predictive value in assessing health risks at all stages of a woman’s life cycle. For example, a shorter reproductive lifespan is thought to be associated with increased risk of osteoporosis [8], hormone-sensitive tumors [9], and cardiovascular disease [10].

Cotinine, the primary metabolite of nicotine, has a biological half-life of approximately 16–19 h [11] and is widely used as a reliable biomarker for assessing tobacco exposure [12]. Studies have shown that cotinine acts on multiple target organs such as the nervous, cardiovascular, renal, hepatic, and reproductive organs [13]. Current research on the effects of tobacco exposure on the female reproductive system, particularly reproductive life span, a composite indicator of ovarian reserve function, relies heavily on self-report of smoking history [14], which is subject to significant recall bias. There is an urgent need to introduce and validate objective indicators, such as cotinine, to improve the accuracy of exposure assessment. In addition, most of the existing studies have used linear models, ignoring possible non-linear or threshold effects between tobacco exposure and reproductive longevity. Notably, factors such as race and socioeconomic status may modulate the toxic effects of tobacco on the reproductive system; however, studies in this area remain limited and have yet to be systematically validated in cross-cultural populations.

To address these knowledge gaps, this study innovatively integrated two nationally representative population databases, the National Health and Nutrition Examination Survey (NHANES) and the Korean National Health and Nutrition Examination Survey (KNHANES), and included 11,944 female participants. The association characteristics between tobacco exposure levels and reproductive life span were systematically assessed by standardized testing of serum cotinine in NHANES and urine cotinine concentrations in KNHANES, using nonlinear modeling methods such as restricted cubic spline. The study further explored the potential role of race, socioeconomic status, and metabolic diseases (e.g., diabetes and hypertension) as modifiers of the association. The findings are expected to deepen our understanding of tobacco reproductive toxicity, provide an evidence base for developing more precise public health interventions, and help achieve the goal of global reproductive health equity.

## Methods

### Study population 1: the NHANES

NHANES (https://www.cdc.gov/nchs/nhanes/about_nhanes.Htm) is a nationally representative, cross-sectional survey conducted by the U.S. National Center for Health Statistics (NCHS) to evaluate the health and nutritional status of adults and children. All participants provided written informed consent, and the protocol was approved by the NCHS Ethics Review Board. For this study, we included 6,081 women from NHANES cycles 1999–2018 [15].

Potential covariates were selected based on prior literature and included demographic factors (age, race/ethnicity [Mexican American, other Hispanic, non-Hispanic White, non-Hispanic Black, and other races], education level, marital status, and poverty income ratio [PIR]) and lifestyle/clinical factors (body mass index [BMI], hypertension, diabetes mellitus, and use of hormone medication or oral contraceptives [OC]). Education was categorized as less than high school, high school graduate/General Educational Development [GED] or equivalent, and higher than high school. Marital status was grouped as married/living with partner or widowed/separated/divorced. PIR was stratified into ≤ 1.3, 1.3–3.5, and > 3.5 [16].

In both NHANES and KNHANES, we restricted the analysis to women who had experienced natural menopause and had available information on age at menarche and age at menopause, which allowed the calculation of reproductive lifespan. Eligible participants were additionally required to have valid biomarker data on serum cotinine (NHANES) or urinary cotinine (KNHANES), as well as complete information on all predefined covariates. Women with surgically induced menopause (e.g., hysterectomy or bilateral oophorectomy) or with age at menarche > 18 years, which may indicate underlying pathology, were not eligible. Participants with missing data for any of these key variables were excluded from the analytic sample.

**Fig. 1:**
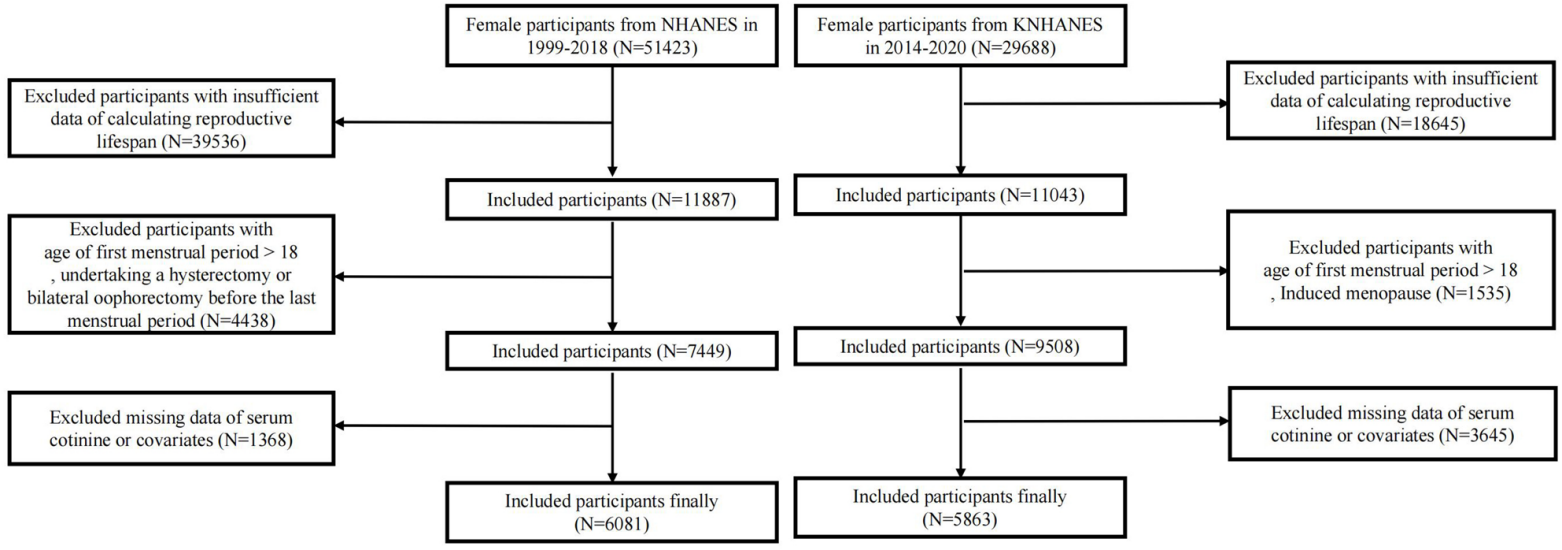
Flowchart of study population selection in NHANES and KNHANES

Of the 51,423 women initially surveyed between 1999 and 2018, we excluded: [1] 39,536 with incomplete data on reproductive lifespan [2], 4,438 with age at menarche > 18 years (suggestive of pathological conditions [16]) or a history of hysterectomy/bilateral oophorectomy, and [3] 1,368 with missing data on serum cotinine or covariates. The final analytic sample comprised 6,081 eligible participants (Fig. 1).

### Study population 2: the KNHANES

The KNHANES has been conducted by the Korea Centers for Disease Control and Prevention (KCDC) since 1998 [17]. All participants provided written informed consent, and the study protocol was approved by the KCDC Research Ethics Review Board.

For the present analysis, we used data from KNHANES cycles 2014–2020, including 5,863 women. Covariates were selected in parallel with NHANES and included demographic characteristics (age, education level, marital status, and poverty income ratio [PIR]) and lifestyle/clinical factors (body mass index [BMI], hypertension, diabetes mellitus, and oral contraceptive [OC] use). PIR was categorized as lower, lower-middle, upper-middle, and upper. Education level and marital status were harmonized with the NHANES definitions for comparability.

Of the 29,688 women initially surveyed during 2014–2020, we excluded: [1] 18,645 with incomplete data on reproductive lifespan [2], 1,535 with age at menarche > 18 years or artificial menopause, and [3] 3,645 with missing exposure or covariate data. The final analytic cohort comprised 5,863 eligible women (Fig. 1).

### Exposure measure

In NHANES, serum cotinine concentrations were quantified using isotope dilution–high-performance liquid chromatography coupled with atmospheric pressure chemical ionization tandem mass spectrometry. In KNHANES, urinary cotinine concentrations were measured on the day of survey completion using gas chromatography–mass spectrometry (PerkinElmer, Waltham, MA, USA) [18].

### Outcome measure

Information on age at menarche and menopause was obtained in part through self-report. Reproductive lifespan (RLS) was defined as the interval between age at menopause and age at menarche [19]. In NHANES, natural menopause was defined after excluding women with a history of bilateral oophorectomy or hysterectomy, whereas in KNHANES, it was determined based on self-reported natural menopause status.

Because urinary cotinine concentrations are influenced by urine dilution, we corrected KNHANES measurements using spot urine creatinine. Specifically, we calculated a cotinine-to-creatinine ratio (ng cotinine per mg creatinine) and used the natural logarithm of this ratio in primary KNHANES analyses as Fig. 4. As a sensitivity analysis, we repeated the models using uncorrected urinary cotinine values, and the direction and magnitude of the associations with reproductive lifespan were materially unchanged.

### Statistical analysis

Analyses were conducted using data from NHANES and KNHANES. For NHANES, all analyses applied sampling weights corresponding to the fasting subsample, as recommended by NHANES guidelines, and estimates were reported in weighted form. Descriptive statistics were generated using t tests for continuous variables and chi-square tests for categorical variables. Continuous variables are presented as mean ± standard deviation, and categorical variables as frequencies and percentages.

Cotinine concentrations were log-transformed to reduce skewness. Multivariable regression models were constructed to evaluate associations between cotinine and reproductive outcomes. Fully adjusted models included age, education, poverty income ratio (PIR), marital status, body mass index (BMI), diabetes, hypertension, and oral contraceptive (OC) use. Restricted cubic spline (RCS) models were further applied, adjusting for the same covariates, to examine potential dose–response relationships. Stratified analyses and multiplicative interaction terms were tested in multivariable models to explore potential effect modification by demographic, socioeconomic, and metabolic factors. All analyses were performed using R software (version 4.3.3; R Foundation for Statistical Computing, Vienna, Austria). A two-sided P value < 0.05 was considered statistically significant.

## Result

### Baseline characteristics

The final analysis included 6,081 NHANES participants and 5,863 KNHANES participants, stratified by cotinine quartiles (Q1–Q4) (Tables 1 and 2). In both cohorts, higher cotinine levels were significantly associated with younger age (NHANES Q4: 50.0% <60 years vs. Q1: 29.2%; KNHANES Q4: 40.5% vs. Q1: 30.1%; both *p* < 0.001). Clear socioeconomic gradients were observed, with participants in the highest quartiles more likely to have lower educational attainment (NHANES: 33.3% vs. 23.4%; KNHANES: 68.4% vs. 63.2%) and greater poverty burden (NHANES PIR ≤ 1.3: 43.3% vs. 23.5%; KNHANES: 30.2% vs. 20.1%). Racial composition differed substantially: in NHANES, Non-Hispanic Black women were disproportionately represented in higher quartiles (Q4: 26.3% vs. Q1: 8.1%, *p* < 0.001), whereas KNHANES showed a relatively uniform ethnic distribution.

**Table 1:** NHANCE database: baseline characteristics

Variables	Total	Serum cotinine, ng/mL				*P*
		Q1 (≤0.011)	Q2 (0.011–0.031.011.031)	Q3 (0.031–0.207.031.207)	Q4 (>0.207)	
n	6081	1927	1134	1501	1519	
Age, n (%)						< 0.001
<60years	2190 (36.0)	563 (29.2)	387 (34.1)	480 (32.0)	760 (50.0)	
≥60years	3891 (64.0)	1364 (70.8)	747 (65.9)	1021 (68.0)	759 (50.0)	
Race, n (%)						< 0.001
Mexican American	988 (16.2)	376 (19.5)	174 (15.3)	256 (17.1)	182 (12.0)	
Other Hispanic	573 (9.4)	229 (11.9)	127 (11.2)	114 (7.6)	103 (6.8)	
Non-Hispanic White	3001 (49.4)	1016 (52.7)	528 (46.6)	697 (46.4)	760 (50.0)	
Non-Hispanic Black	1040 (17.1)	157 (8.1)	190 (16.8)	294 (19.6)	399 (26.3)	
Other Race - Including Multi-Racial	479 (7.9)	149 (7.7)	115 (10.1)	140 (9.3)	75 (4.9)	
Education, n (%)						< 0.001
Less than high school	1737 (28.6)	450 (23.4)	293 (25.8)	488 (32.5)	506 (33.3)	
High school grad/GED or equivalent	1478 (24.3)	402 (20.9)	258 (22.8)	381 (25.4)	437 (28.8)	
Higher than high school	2866 (47.1)	1075 (55.8)	583 (51.4)	632 (42.1)	576 (37.9)	
PIR, n (%)						< 0.001
≤1.3	1906 (31.3)	453 (23.5)	301 (26.5)	495 (33.0)	657 (43.3)	
1.3–3.5.3.5	2329 (38.3)	728 (37.8)	434 (38.3)	592 (39.4)	575 (37.9)	
>3.5	1846 (30.4)	746 (38.7)	399 (35.2)	414 (27.6)	287 (18.9)	
Marital status, n (%)						< 0.001
Married/living with partner	3067 (50.4)	1074 (55.7)	615 (54.2)	727 (48.4)	651 (42.9)	
Widowed/divorced/separated	2541 (41.8)	725 (37.6)	453 (39.9)	678 (45.2)	685 (45.1)	
Never married	473 (7.8)	128 (6.6)	66 (5.8)	96 (6.4)	183 (12.0)	
BMI, n (%)						< 0.001
<25 kg/m2	1678 (27.6)	558 (29.0)	288 (25.4)	366 (24.4)	466 (30.7)	
25–30 kg/m2	1902 (31.3)	629 (32.6)	343 (30.2)	490 (32.6)	440 (29.0)	
≥30 kg/m2	2501 (41.1)	740 (38.4)	503 (44.4)	645 (43.0)	613 (40.4)	
Diabetes, n (%)						0.683
Yes	1052 (17.3)	320 (16.6)	204 (18.0)	269 (17.9)	259 (17.1)	
No	5029 (82.7)	1607 (83.4)	930 (82.0)	1232 (82.1)	1260 (82.9)	
Hypertension, n (%)						0.127
Yes	3093 (50.9)	960 (49.8)	564 (49.7)	803 (53.5)	766 (50.4)	
No	2988 (49.1)	967 (50.2)	570 (50.3)	698 (46.5)	753 (49.6)	
OC use, n (%)						< 0.001
Yes	3586 (59.0)	1138 (59.1)	665 (58.6)	793 (52.8)	990 (65.2)	
No	2495 (41.0)	789 (40.9)	469 (41.4)	708 (47.2)	529 (34.8)	
Hormone use, n (%)						< 0.001
Yes	1715 (28.2)	620 (32.2)	355 (31.3)	424 (28.2)	316 (20.8)	
No	4366 (71.8)	1307 (67.8)	779 (68.7)	1077 (71.8)	1203 (79.2)	< 0.001
Menarche age, [years, mean (SD)]	12.902 (1.748)	12.862 (1.685)	12.869 (1.674)	13.032 (1.814)	12.851 (1.808)	0.012
Menopausal age, [years, mean (SD)]	47.347 (7.722)	48.416 (6.661)	47.944 (6.945)	47.891 (7.462)	45.009 (9.168)	< 0.001
Reproductive lifespan, [years, mean (SD)]	34.445 (7.849)	35.554 (6.838)	35.075 (7.075)	34.859 (7.627)	32.159 (9.236)	< 0.001

Q1-Q4: Quartile 1 to Quartile 4;GED: General Educational Development; PIR: Poverty-income ratio; BMI: Body mass index; OC: Oral Contraceptive; SD: Standard deviation

**Table 2:** KNHANES database: baseline characteristics

Variables	Total	Urine cotinine, ng/mL				*P*
		Q1 (≤0.401)	Q2 (0.401–0.7.401.7)	Q3 (0.7–1.625.7.625)	Q4 (>1.625)	
n	5863	1466	1468	1463	1466	
Age, n (%)						< 0.001
<60years	2064 (35.2)	441 (30.1)	490 (33.4)	539 (36.8)	594 (40.5)	
≥60years	3799 (64.8)	1025 (69.9)	978 (66.6)	924 (63.2)	872 (59.5)	
Education, n (%)						< 0.001
Less than high school	3808 (64.9)	927 (63.2)	941 (64.1)	937 (64.0)	1003 (68.4)	
High school grad	1403 (23.9)	346 (23.6)	332 (22.6)	368 (25.2)	357 (24.4)	
Higher than high school	652 (11.1)	193 (13.2)	195 (13.3)	158 (10.8)	106 (7.2)	
PIR, n (%)						< 0.001
Low	1374 (23.4)	295 (20.1)	300 (20.4)	337 (23.0)	442 (30.2)	
Lower-middle	1490 (25.4)	366 (25.0)	369 (25.1)	366 (25.0)	389 (26.5)	
Upper-middle	1496 (25.5)	359 (24.5)	398 (27.1)	397 (27.1)	342 (23.3)	
High	1503 (25.6)	446 (30.4)	401 (27.3)	363 (24.8)	293 (20.0)	
Marital status, n (%)						0.001
Married/living with partner	3983 (67.9)	1025 (69.9)	990 (67.4)	1029 (70.3)	939 (64.1)	
Widowed/divorced/separated	1880 (32.1)	441 (30.1)	478 (32.6)	434 (29.7)	527 (35.9)	
BMI, n (%)						0.851
<25 kg/m^2^	3733 (63.7)	945 (64.5)	934 (63.6)	925 (63.2)	929 (63.4)	
25–30 kg/m^2^	1820 (31.0)	446 (30.4)	462 (31.5)	450 (30.8)	462 (31.5)	
≥30 kg/m^2^	310 (5.3)	75 (5.1)	72 (4.9)	88 (6.0)	75 (5.1)	
Diabetes, n (%)						0.195
Yes	812 (13.8)	195 (13.3)	190 (12.9)	200 (13.7)	227 (15.5)	
No	5051 (86.2)	1271 (86.7)	1278 (87.1)	1263 (86.3)	1239 (84.5)	
Hypertension, n (%)						0.228
Yes	2299 (39.2)	590 (40.2)	592 (40.3)	574 (39.2)	543 (37.0)	
No	3564 (60.8)	876 (59.8)	876 (59.7)	889 (60.8)	923 (63.0)	
OC use, n (%)						0.302
Yes	1344 (22.9)	314 (21.4)	334 (22.8)	339 (23.2)	357 (24.4)	
No	4519 (77.1)	1152 (78.6)	1134 (77.2)	1124 (76.8)	1109 (75.6)	
Menarche age, [years, mean (SD)]	14.948 (1.759)	14.945 (1.729)	14.973 (1.769)	14.927 (1.746)	14.948 (1.790)	0.917
Menopausal age, [years, mean (SD)]	49.912 (4.244)	50.054 (4.213)	50.053 (4.138)	49.963 (4.210)	49.579 (4.398)	0.006
Reproductive lifespan, [years, mean (SD)]	34.964 (4.611)	35.109 (4.550)	35.080 (4.554)	35.036 (4.575)	34.631 (4.750)	0.015

Q1-Q4: Quartile 1 to Quartile 4; PIR: Poverty-income ratio; BMI: Body mass index; OC: Oral Contraceptive; SD: Standard deviation

Reproductive health indicators demonstrated consistent dose–response associations. Higher cotinine levels were linked to reduced hormone therapy use (NHANES Q4: 20.8% vs. Q1: 32.2%, *p* < 0.001), earlier menopausal age (NHANES: 45.0 vs. 48.4 years; KNHANES: 49.6 vs. 50.1 years), and shorter reproductive lifespan (NHANES: 32.2 vs. 35.6 years; KNHANES: 34.6 vs. 35.1 years). No significant associations were detected with metabolic conditions, including diabetes and hypertension (*p* > 0.05). BMI distributions varied significantly across cotinine quartiles in NHANES (*p* < 0.001) but not in KNHANES (*p* = 0.851). Patterns of oral contraceptive use diverged between cohorts (NHANES Q4: 65.2% vs. Q1: 59.1%, *p* < 0.001; KNHANES *p* = 0.302). Marital status distributions differed significantly in both cohorts (NHANES *p* < 0.001; KNHANES *p* = 0.001). Age at menarche remained relatively stable across quartiles in both populations.

### Results of linear regression analysis

Linear regression analyses (Table 3) demonstrated significant associations between log-transformed cotinine (ln-cotinine) levels and reproductive aging parameters. For age at menarche, a modest inverse association was observed in KNHANES (β = −0.02, 95% CI: −0.04 to 0.00, *p* = 0.027), whereas no significant association was found in NHANES (*p* = 0.144).

**Table 3:** Association of Serum Cotinine With Reproductive Aging: Linear Regression Analysis

Variables	Cotinine	NHANCE(Serum)		KNHANES(Urine)	
		β (95% CI)	P	β (95% CI)	P
Menarche age	Ln-cotinine	−0.01(−0.02, 0.00)	0.144	−0.02(−0.04, 0.00)	0.027
	Q1	Reference		Reference	
	Q2	0 (−0.12, 0.13)	0.971	0.04 (−0.08, 0.16)	0.515
	Q3	0.12 (0.00, 0.23)	0.051	0 (−0.11, 0.12)	0.96
	Q4	−0.02 (−0.14, 0.10)	0.767	−0.02 (−0.14, 0.10)	0.719
	P for trend	0.415		0.526	
Menopausal age	Ln-cotinine	−0.23 (−0.29, −0.18)	< 0.001	−0.1 (−0.15, −0.05)	< 0.001
	Q1	Reference		Reference	
	Q2	−0.28 (−0.81, 0.26)	0.311	0.03 (−0.28, 0.33)	0.865
	Q3	−0.21 (−0.70, 0.29)	0.413	−0.07 (−0.38, 0.24)	0.662
	Q4	−2 (−2.5, −1.5)	< 0.001	−0.37 (−0.68, −0.06)	0.02
	P for trend	< 0.001		0.008	
Reproductive lifespan	Ln-cotinine	−0.22 (−0.28, −0.17)	< 0.001	−0.08 (−0.13, −0.02)	0.011
	Q1	Reference		Reference	
	Q2	−0.28 (−0.82, 0.27)	0.317	−0.01 (−0.34, 0.32)	0.94
	Q3	−0.32 (−0.83, 0.18)	0.211	−0.07 (−0.40, 0.26)	0.67
	Q4	−1.98 (−2.5, −1.45)	< 0.001	−0.35 (−0.68, −0.01)	0.041
	P for trend	< 0.001		0.024	

All models were adjusted for age, education, PIR, marital status, BMI, diabetes, hypertension, and OC use. The "P for trend" value assesses the presence of a statistically significant dose-response relationship across ordered categories (quartiles Q1–Q4 of cotinine levels). β represents the regression coefficient. CI: 95% confidence interval. PIR: Poverty-income ratio; BMI: Body mass index; OC:Oral Contraceptive

In contrast, both cohorts revealed strong dose–response relationships between higher ln-cotinine levels and earlier age at menopause (NHANES: β = −0.23, 95% CI: −0.29 to − 0.18, *p* < 0.001; KNHANES: β = −0.10, 95% CI: −0.15 to − 0.05, *p* < 0.001), with particularly pronounced effects in the highest quartile (NHANES Q4: β = −2.00, *p* < 0.001; KNHANES Q4: β = −0.37, *p* = 0.020). Similarly, reproductive lifespan (RLS) showed significant inverse associations with ln-cotinine (NHANES: β = −0.22, 95% CI: −0.28 to − 0.17, *p* < 0.001; KNHANES: β = −0.08, 95% CI: −0.13 to − 0.02, *p* = 0.011), again with the strongest effects in Q4 (NHANES: β = −1.98, *p* < 0.001; KNHANES: β = −0.35, *p* = 0.041). Trend analyses confirmed statistically significant inverse associations for both age at menopause and RLS across quartiles in both cohorts (all *p* < 0.05).

### Subgroup analysis

**Fig. 2:**
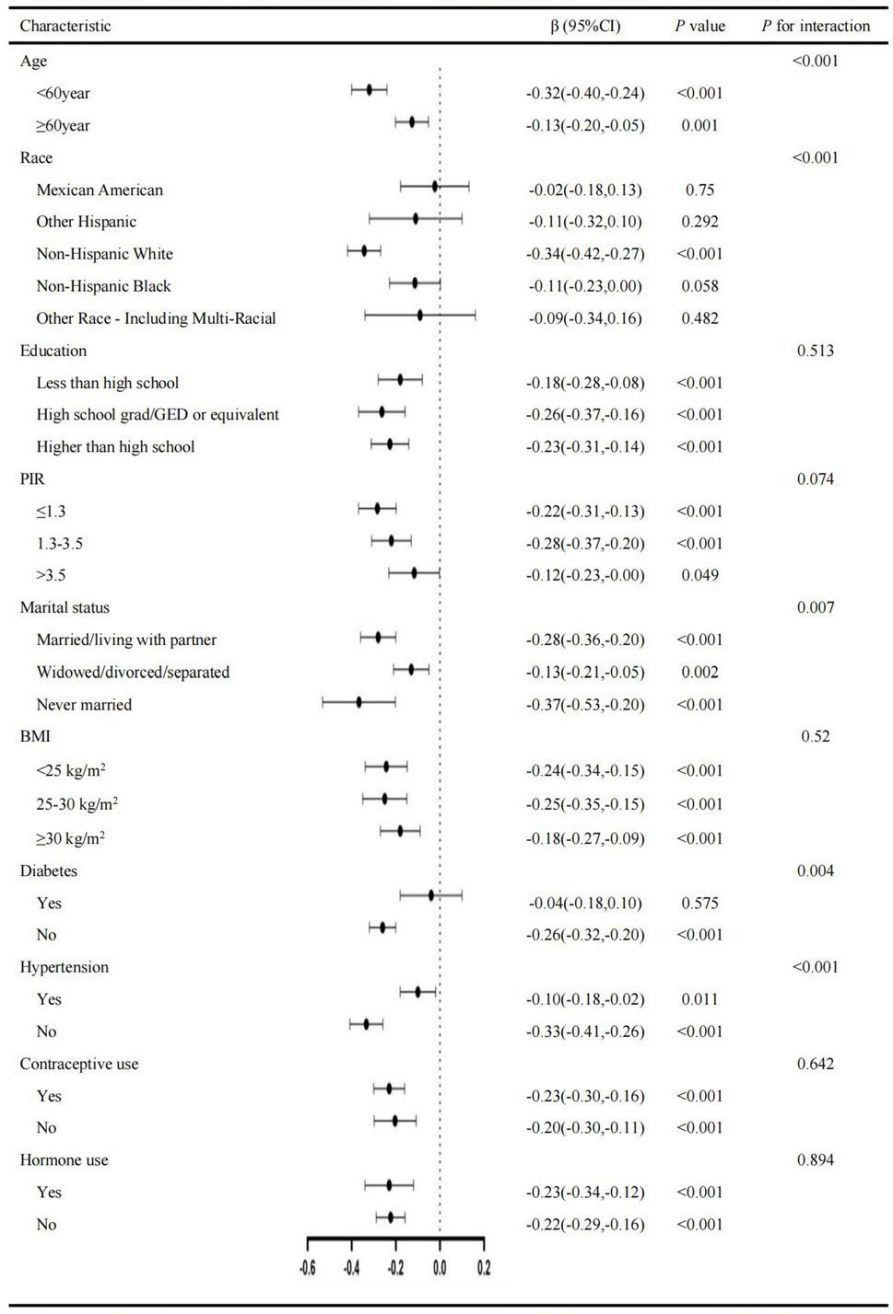
Subgroup analyses of the association between ln-cotinine levels and RLS in NHANES. Forest plots show multivariable linear regression estimates (β coefficients with 95% CIs) stratified by age, diabetes, hypertension, poverty income ratio (PIR), race/ethnicity, education, marital status, BMI, oral contraceptive (OC) use, and hormone use. Stronger negative associations were observed among younger, non-diabetic, non-hypertensive, and low-income participants

**Fig. 3:**
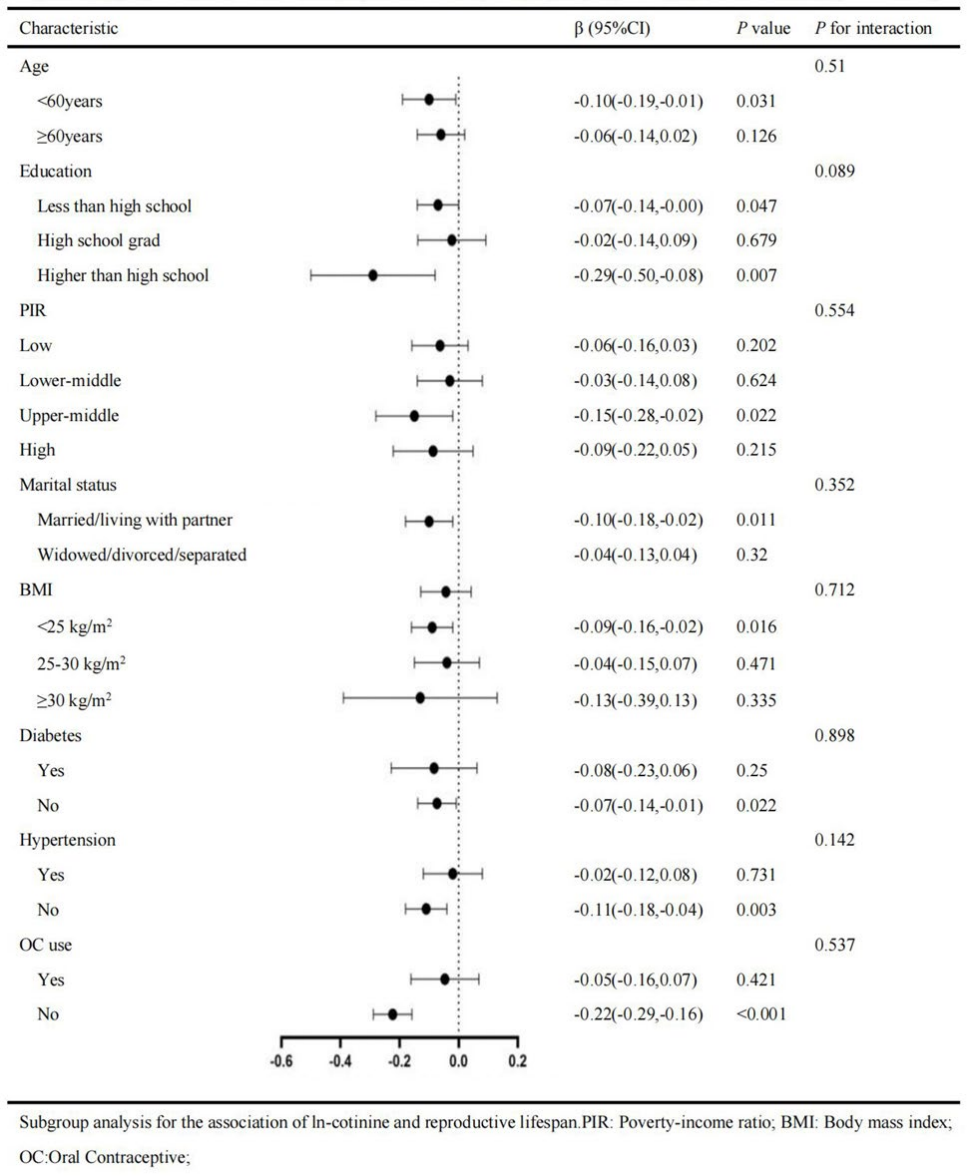
Subgroup analyses of the association between ln-cotinine levels and RLS in KNHANES. Forest plots display regression estimates (β coefficients with 95% CIs) stratified by age, diabetes, hypertension, PIR, education, marital status, BMI, and OC use. More pronounced associations were observed among younger, non-diabetic women, and those with higher educational attainment or without OC use

Subgroup analyses (Figs. 2 and 3) revealed consistent inverse associations between ln-cotinine levels and RLS across both cohorts, with notable heterogeneity in effect sizes. Stronger associations were observed among younger participants (< 60 years: NHANES β = −0.32 vs. KNHANES β = −0.10) and non-diabetic individuals (NHANES β = −0.26 vs. KNHANES β = −0.07; both *p* < 0.05). Socioeconomic gradients were evident in NHANES, where the effect size attenuated progressively from low-income (PIR ≤ 1.3: β = −0.22) to high-income groups (PIR > 3.5: β = −0.12, p for interaction = 0.074). In contrast, KNHANES showed no significant PIR-related differences (p for interaction = 0.554). Hypertension status significantly modified the associations in NHANES (non-hypertensive β = −0.33 vs. hypertensive β = −0.10, p for interaction < 0.001), but not in KNHANES (*p* = 0.142). Racial disparities were pronounced in NHANES, where Non-Hispanic White participants demonstrated the strongest association (β = −0.34, *p* < 0.001), while race-stratified analyses were unavailable in KNHANES.

Divergent patterns were also observed for education and marital status. In NHANES, associations were consistent across education levels (p for interaction = 0.513), whereas in KNHANES, stronger effects were observed among women with higher education (β = −0.29, *p* = 0.007). Marital status significantly modified the associations in NHANES (never-married: β = −0.37, *p* < 0.001), but not in KNHANES (p for interaction = 0.352). OC use modified the association exclusively in KNHANES, where stronger effects were seen among non-users (β = −0.22, *p* < 0.001). BMI strata showed no significant interactions in either cohort (*p* > 0.5). Hormone use did not significantly modify the associations in NHANES (p for interaction = 0.894).

### RCS analysis

**Fig. 4:**
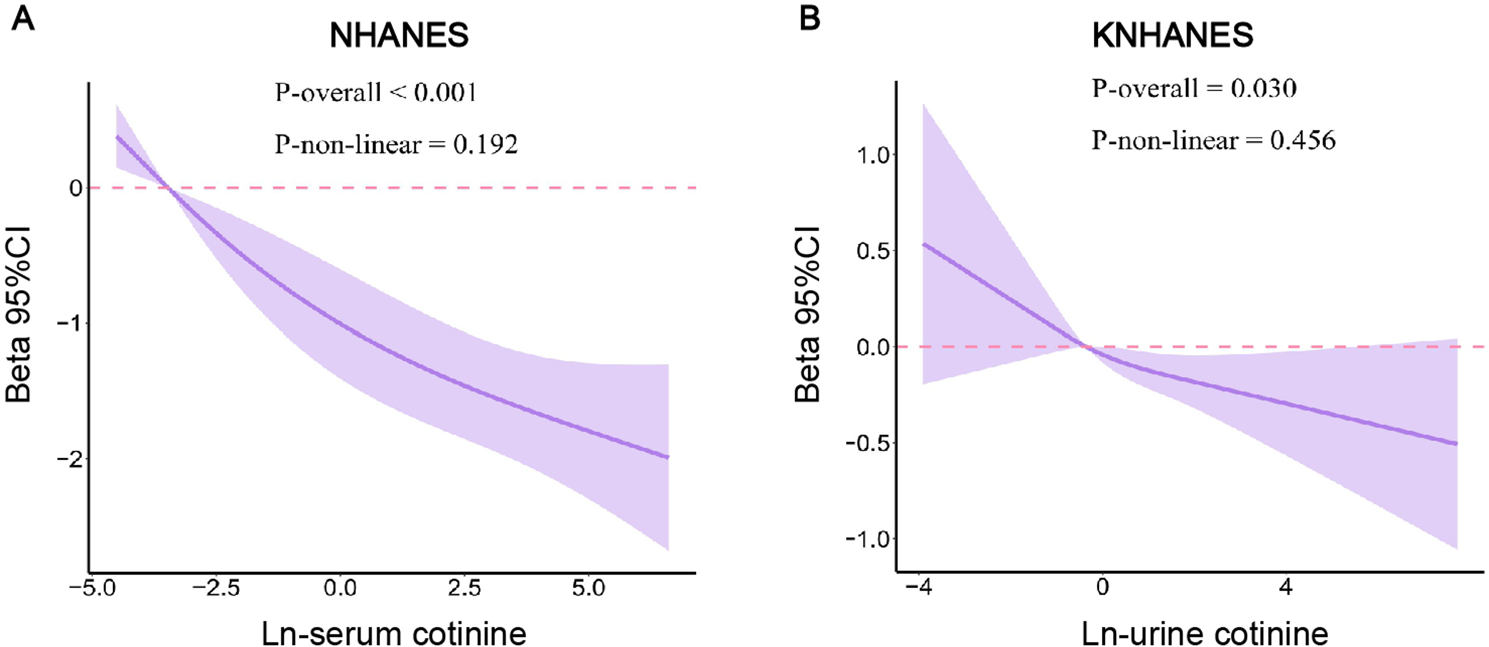
Restricted cubic spline (RCS) analyses of ln-cotinine and reproductive lifespan (RLS) in NHANES and KNHANES.(A) NHANES: fully adjusted RCS model for the association between ln-serum cotinine and RLS. (B) KNHANES: fully adjusted RCS model for the association between ln-urine cotinine and RLS. Higher cotinine exposure was associated with shorter RLS in both cohorts, with no evidence of non-linearity (P = 0.192 in NHANES; 0.456 in KNHANES)

Restricted cubic spline analyses (Fig. 4) confirmed linear dose–response relationships between ln-cotinine levels and RLS in both cohorts (NHANES *p* < 0.001; KNHANES *p* = 0.030), with no evidence of nonlinearity (p for nonlinearity ≥ 0.192). Restricted cubic spline models with three knots placed at the 10th, 50th, and 90th percentiles of ln-cotinine were used to flexibly assess dose–response relationships, and sensitivity analyses using alternative knot locations produced similar results. Threshold analyses (Table 4) indicated that in NHANES, higher ln-cotinine levels (> − 3.47) were significantly associated with reduced RLS (β = −0.303, 95% CI: −0.386 to − 0.220, *p* < 0.001). In contrast, KNHANES demonstrated only a non-significant trend toward decreased RLS at elevated ln-cotinine levels (> − 0.356; β = −0.059, 95% CI: −0.135 to 0.017, *p* = 0.127).

**Table 4:** Dose-Response relationships between Ln-Cotinine and reproductive lifespan with threshold effects in NHANES and KNHANES cohorts

NHANES			KNHANES		
Ln-cotinine	β (95%CI)	*P*	Ln-cotinine	β (95%CI)	*P*
≤−3.47	−0.607 (−1.318, −0.105)	0.095	≤−0.356	−0.133 (−0.426, 0.160)	0.373
>−3.47	−0.303(−0.386, −0.220)	< 0.001	>−0.356	−0.059 (−0.135, 0.017)	0.127
Log likelihood ratio test		0.562	Log likelihood ratio test		0.593

In both cohorts, higher ln-cotinine levels were associated with shorter reproductive lifespan in a generally linear fashion. Exploratory segmented models suggested slightly steeper slopes at higher exposure levels; however, formal likelihood ratio tests for threshold effects were not statistically significant (NHANES *p* = 0.562; KNHANES *p* = 0.593).

## Discussion

This study systematically evaluated the negative association between smoking exposure, measured by cotinine levels, and female reproductive health for the first time in a cross-cultural cohort (NHANES in the United States and KNHANES in Korea). Core findings consistently showed that higher cotinine levels were significantly associated with shorter RLS in fully adjusted models (NHANES β = −0.22; KNHANES β = −0.08). Restricted cubic spline analyses confirmed that the association was linear in both cohorts (P-nonlinearity ≥ 0.192). The dose–response threshold effect suggests that adverse impacts on RLS occur only at high levels of exposure, particularly among U.S. women. These findings indicate that cotinine, as an objective biomarker of smoking exposure, is significantly associated with shorter RLS in women, with this effect being especially pronounced at higher exposure levels.

Cotinine, a major metabolite of nicotine, is an important biomarker for assessing tobacco exposure [11]. A growing body of research suggests that cotinine impairs ovarian function through multiple biological mechanisms, which in turn accelerate female reproductive aging and interact with sociodemographic characteristics, ultimately leading to a shorter RLS. Firstly, regarding ovarian hormone synthesis, in vitro studies have found that cigarette smoke extract and its alkaloids (such as nicotine, cotinine, and anabasine) can inhibit progesterone production in human granulosa cells and luteal cells [20, 21]. Moreover, cotinine may be achieved through inhibition of aromatase activity, which reduces the conversion of androstenedione to estrogen, leading to blockage of estrogen synthesis [22–24]. A decline in progesterone levels during the luteal phase may weaken the negative feedback on follicle-stimulating hormone(FSH), leading to elevated FSH levels during the luteal–follicular transition in smokers. This, in turn, may promote earlier follicle recruitment and result in a shortened follicular phase [25]. This change of elevated FSH and shortened follicular phase is thought to characterize accelerated ovarian follicular recruitment and depletion, and is highly correlated with early menopause and reproductive aging [26, 27]. Cotinine was also significantly and negatively associated with anti-mullerian hormone (AMH) levels [28]. In one study, smokers were found to have significantly lower mean AMH concentrations in follicular fluid than non-smokers, suggesting a decrease in ovarian reserve function, which leads to reproductive aging [29]. Cotinine accelerates the depletion of ovarian reserve through multiple mechanisms, including inhibiting steroid hormone synthesis, disrupting endocrine feedback loops, affecting FSH secretion patterns, and decreasing AMH levels, which together lead to accelerated ovarian reserve depletion, manifested by an earlier age at menopause (NHANES Q4:45.0 vs. Q1:48.4 years) and a shorter reproductive life span (NHANES:32.2 vs. 35.6 years). Notably, this reproductive toxicity showed a clear dose-response relationship, with the most potent effects in the highest exposure group (Q4) (NHANES β=−2.0; KNHANES β=−0.37) and a threshold effect, with significant harms only seen when cotinine exceeded a critical concentration.

Such biological damage may interact with socioeconomic disadvantage, creating a self-reinforcing vicious cycle. The high cotinine population is characterized by youthfulness (NHANES Q4:50% <60 years), low educational attainment (NHANES:33.3%), and high rates of poverty (PIR ≤ 1.3:43.3%), which combine to exacerbate reproductive health risks. Low socioeconomic status (SES) populations are more likely to maintain high levels of tobacco use over time and have difficulty quitting [30], leading to continued high levels of cotinine exposure, while limited access to healthcare resources in low SES populations, as evidenced by significantly lower rates of hormone replacement therapy utilization (NHANES Q4:20.8% vs. Q1:32.2%), further exacerbating the adverse effects of estrogen deficiency on the reproductive system [31]. Of particular note, this damage is reproductive system specific and not significantly associated with metabolic diseases such as diabetes/hypertension (*p* > 0.05), suggesting that cotinine may preferentially act directly on ovarian tissue through multiple mechanisms such as inhibition of steroid hormone synthesis, disruption of endocrine feedback loops, influencing FSH secretion patterns, and lowering AMH levels, and that patients with diabetes/hypertension may be at risk due to disease management needs for earlier health interventions (e.g., smoking cessation), resulting in a partially masked negative association between their cotinine levels and reproductive life span. The adverse effect of cotinine on reproductive life span was more pronounced in the nondiabetic population (NHANES β = −0.26). In contrast, the association was weakened in diabetic patients, possibly because chronic inflammation and endocrine disruption caused by diabetes already interfere more strongly with reproductive function, thus weakening the independent effect of cotinine in the statistical model. Together, these findings reveal a complex mechanism by which cotinine accelerates the process of female reproductive aging through the interplay of direct biological toxicity and socio-environmental factors.

The present study revealed significant cross-cultural differences in the association of cotinine with reproductive longevity (NHANES β=−0.22 vs. KNHANES β=−0.08), possibly because of cohort differences in the type of samples tested for cotinine (serum cotinine for NHANES and urine cotinine for KNHANES), with serum cotinine having a longer half-life (approximately 16–20 h) [11] that better reflects long-term tobacco exposure levels. In contrast, urinary cotinine is influenced by recent intake and renal function [32], a difference that may partially explain the observed differences in effect strength. In terms of population heterogeneity, the significant poverty rate gradient in the United States (β=−0.22 in the PIR ≤ 1.3 group vs. β=−0.12 in the > 3.5 group) was directly associated with unequal access to healthcare resources, which was buffered by the relatively homogeneous universal healthcare system in South Korea (interaction *p* = 0.554). The most substantial effect in non-Hispanic whites in the NHANES (β=−0.34) may involve differences in nicotine metabolism due to polymorphisms in the CYP2A6 gene [33]. This genetic factor was not observed in the homogenized Korean population. Despite differences in sample type, both cohorts used standardized laboratory tests, and both restricted cubic spline analyses showed linear dose-response relationships (P nonlinear ≥ 0.192), supporting the veracity of the biological associations. Based on these findings, we recommend the implementation of culturally appropriate precision interventions: community-based smoking cessation programs integrating medical assistance should be targeted to ethnic minorities and low-income groups in the United States, whereas health literacy needs to be reinforced through the education system in South Korea (β=−0.29 effect for the high-education group). Future studies should use a uniform sample type or convert across substrate concentrations through metabolic kinetic modeling to enhance the comparability of results. The core finding of this study-that high levels of cotinine exposure significantly shorten reproductive life span-was validated in both testing systems, providing a scientific basis for developing differentiated public health strategies.

This study has important innovative values at both methodological and theoretical levels. In terms of methodology, the study adopted the biomarker detection technology of dual nationally representative cohorts (NHANES and KNHANES), and overcame the recall bias of traditional questionnaires by quantitative analysis of cotinine to realize the precise quantification of tobacco exposure. The innovative use of restricted cubic spline analysis not only confirmed the linear dose-response relationship between cotinine and reproductive life span (P non-linearity ≥ 0.192), but also identified the critical exposure threshold for the first time, which provides an important basis for the development of public health safety standards. At the theoretical level, the study made a breakthrough finding: after excluding the confounding effects of metabolic diseases such as hypertension and diabetes mellitus (*p* > 0.05), the independent detrimental effect of cotinine on RLS was even more pronounced (β=−0.26 in the nondiabetic group), which is of great clinical value and suggests that for women with normal metabolic indexes, direct toxicity of tobacco exposure on the reproductive system should be taken seriously. Especially importantly, despite the difference in effect sizes between the two cohorts (β=−0.22 vs. −0.08), the consistency of the linear associations and the cross-cultural reproducibility of the threshold effects provide strong support for the generalizability of the findings. These innovative findings expand the theoretical knowledge of the relationship between environmental exposures and reproductive aging and provide a scientific basis for developing precise reproductive health protection strategies in different cultural contexts.

The mechanistic pathways discussed (e.g., inhibition of aromatase, altered FSH dynamics, reduced AMH) are based on prior experimental and clinical studies and were not directly evaluated in our analyses; they should therefore be viewed as plausible biological explanations rather than confirmed causal mechanisms in this dataset. Beyond matrix type and healthcare system differences, the heterogeneity between NHANES and KNHANES may also reflect variation in genetic polymorphisms involved in nicotine metabolism (such as CYP2A6 and UGT2B10), culturally specific smoking patterns, and differing prevalence of passive smoke exposure, all of which warrant more systematic investigation in future work.

Despite the large, nationally representative samples and standardized biomarker assays, several limitations should be noted. First, the cross-sectional design of NHANES and KNHANES precludes causal inference, and a single cotinine measurement with a relatively short elimination half-life primarily reflects recent rather than cumulative tobacco exposure, raising the possibility of exposure misclassification and reverse causation (e.g., women who reached menopause earlier may subsequently reduce or quit smoking) [34]. Second, a large proportion of women were excluded due to missing reproductive information, which may have introduced selection bias and limited generalizability to participants with more complete data. Third, cotinine was measured in different matrices (serum in NHANES and urine in KNHANES), and reproductive lifespan was derived from self-reported ages at menarche and menopause; matrix-related differences and recall error, together with the absence of direct ovarian reserve or hormonal biomarkers (e.g., AMH, FSH, antral follicle count), may have led to non-differential exposure and outcome misclassification and attenuation of true associations. Fourth, residual confounding from unmeasured lifestyle, reproductive, and environmental factors (such as parity, alcohol intake, occupational exposures, passive smoking, and psychological stress) cannot be excluded despite multivariable adjustment. Finally, our mechanistic interpretations and any apparent exposure “thresholds” are speculative, as no hormonal or genetic markers were measured in parallel with cotinine and formal tests for threshold effects were not statistically significant; these patterns should therefore be regarded as exploratory. Future research should prioritize longitudinal cohort studies with repeated cotinine measurements, detailed smoking histories, and objective biomarkers of ovarian aging, ideally integrated with multi-omics approaches, to clarify temporal relationships and underlying mechanisms. Interventional studies evaluating the impact of smoking cessation on reproductive lifespan and risk-prediction models that combine clinical indicators with biomarker data will be essential to refine dose–response estimates and support individualized reproductive health protection strategies.

## Conclusion

In this cross-cultural analysis of NHANES and KNHANES, higher cotinine levels were modestly associated with shorter reproductive lifespan, with more consistent effects observed in the U.S. cohort. Restricted cubic spline analyses supported generally linear associations, although evidence for threshold effects was limited and appeared more apparent at higher exposure levels. While cotinine may serve as a useful objective biomarker of tobacco exposure in studies of reproductive aging, these findings should be interpreted with caution given cohort differences and the observational design. Further longitudinal and mechanistic research is warranted to clarify the extent and pathways of these associations.

## Data Availability

The survey data are publicly available on the internet for data users and researchers throughout the world.

## Abbreviations

AMH: Anti-mullerian hormone
BMI: Body mass index
FSH: Follicle-stimulating hormone
GED: General Educational Development
KCDC: The Korea Centers for Disease Control and Prevention
KNHANES: The Korea National Health and Nutrition Examination Survey
NCHS: The National Center for Health Statistics
NHANES: The National Health and Nutrition Examination Survey
OC: Oral Contraceptive
PIR: Poverty Income Ratio
RLS: Reproductive lifespan
RCS: Restricted cubic spline
SES: Low socioeconomic status
